# Relationship between periodontal status of mandibular incisors and selected cephalometric parameters: Preliminary results

**DOI:** 10.1007/s00056-019-00170-0

**Published:** 2019-03-01

**Authors:** Edyta Kalina, Małgorzata Zadurska, Ewa Sobieska, Bartłomiej Górski

**Affiliations:** 1 Department of Orthodontics, Medical University of Warsaw, Nowogrodzka St 59, 02-006 Warsaw, Poland; 2 Department of Periodontology and Oral Diseases, Medical University of Warsaw, Miodowa St 18, 00-246 Warsaw, Poland

**Keywords:** Mandibular incisors, Gingival phenotype, Periodontal phenotype, Skeletal morphology, Cephalometry, Untere Schneidezähne, Gingivaler Phänotyp, Parodontaler Phänotyp, Skelettale Morphologie, Kephalometrie

## Abstract

**Purpose:**

The aim of this cross-sectional study was to evaluate the correlation between the periodontal tissue of mandibular incisors and several dentoalveolar and skeletal cephalometric parameters.

**Materials and methods:**

The sample consisted of 35 patients (mean age 26.42 ± 8.02 years). Eligibility criteria included good overall health status with no history of dental trauma, congenital defects, active periodontal diseases, restorative and prosthetic treatment in the area of the mandibular incisors. Gingival recession width and height (GRW, GRH), gingival thickness (GT), width of keratinized gingiva (WKT) and clinical attachment loss (CAL) were evaluated at 140 lower incisors. Incisors inclination (1-:ML), skeletal class (ANB, WITS), intermaxillary angle (NL:ML) and mandibular symphysis dimensions (symph. length and width) were assessed in cephalograms. Spearman’s correlation coefficient was used for statistical analysis at the *P* < 0.05 level.

**Results:**

A statistically significant positive moderate correlation was found for GT and WITS and also symph. length. WKT correlated positively with ANB, WITS and symph. length, with moderate strength of the correlation. GRW, GRH and CAL did not correlate with any cephalometric parameters.

**Conclusion:**

The results of this study indicated evidence for an association between WKT and GT and some cephalometric variables—ANB, WITS, and symphysis length.

## Zusammenfassung

**Ziel:**

Ziel der vorliegenden Querschnittsstudie war es, den Zusammenhang zwischen dem parodontalen Zustand unterer Schneidezähne und verschiedenen dentoalveolären und skelettalen kephalometrischen Parametern zu untersuchen.

**Material und Methoden:**

Das Patientengut bestand aus 35 Probanden (durchschnittliches Alter: 26,4 ± 8,0 Jahre). Zu den Auswahlkriterien gehörten ein guter Allgemeinzustand ohne Zahntrauma in der Vergangenheit, keine angeborene Defekte, keine aktive Parodontalerkrankung sowie restaurative und prothetische Behandlungen im Bereich der Unterkieferschneidezähne. Bei 140 unteren Schneidezähnen wurden Breite und Höhe von Rezessionen (GRW, GRH), Gingivadicke (GT), Breite der keratinisierten Gingiva (WKT) und klinischer Attachmentverlust (CAL) bestimmt. In den Fernröntgenseitenaufnahmen wurden die Inklination der unteren Schneidezähne (1:ML), die skelettale Klasse (ANB, WITS), der Intermaxillarwinkel (NL:ML) und die Dimensionen der Symphyse (Länge und Breite) bewertet. Zur statistischen Analyse diente der Korrelationskoeffizient nach Spearman (Signifikanzniveau: *P* < 0,05).

**Ergebnisse:**

Eine statistisch signifikante positive moderate Korrelation wurde für GT und WITS sowie für die Länge der Symphyse gefunden. WKT korrelierte mäßig positiv mit ANB, WITS und Symphysenlänge. GRW, GRH und CAL korrelierten nicht mit kephalometrischen Parametern.

**Sclussfolgerungen:**

Die Ergebnisse dieser Studie zeigten Anzeichen für einen Zusammenhang zwischen WKT und GT und einigen kephalometrischen Variablen – ANB, WITS und Symphysenlänge.

## Introduction

The orthodontic teeth movement mechanism is based on the remodeling of bone tissue. In order to achieve a stable and esthetically pleasant effect of orthodontic treatment, hard and soft tissues surrounding teeth should be preserved [36]. The limits of orthodontic tooth movement are set by the cortical bone of the alveolar process. Moving the tooth beyond the bone envelope may lead to iatrogenic sequelae—root resorption, dehiscence, and gingival recession [15, 40]. The evaluation of bone support in the area of mandibular incisors is crucial in planning orthodontic treatment. It has been proven that these teeth are most prone to gingival recession [2]. Furthermore, they are often subjected to meaningful position changes during the therapy.

The morphology of mandibular anterior alveolus differs in hypodivergent, hyperdivergent, and norm divergent patients. The dentoalveolar compensatory mechanism results in elongated and narrow process in high-angle patients, whereas in low-angle patients it is short and wide [9]. Interestingly, Molina-Berlanga et al. [26] reported symphysis elongation in class III patients with normal vertical face dimensions. Also the position of the lower incisors is influenced by the vertical skeletal pattern and anterior–posterior discrepancies. In class II patients these teeth are proclined in order to decrease the overbite, whereas in class III patients they are retroclined to minimalize negative overbite [12]. Also in excess vertical growth, dentoalveolar compensation results in upright and frequently overcrowded lower incisors [13, 38].

The gingival phenotype, which can be classified as thick or thin, has been suggested as a modulator of gingival recession following orthodontic treatment [17]. It has been widely implied that when gingival thickness (GT) is >1 mm, the phenotype can be classified as thick, while the thin phenotype is ≤1 mm [16]. The thick phenotype is characterized by dense and fibrotic soft tissue with a large amount of attachment, while the thin phenotype is characterized by delicate soft tissue with a minimal amount of attachment. Being susceptible to trauma and inflammation, the thin phenotype might promote gingival recession. Hence, GT is regarded as an important factor in the planning of orthodontic treatment. Melsen and Allais [25] reported significant correlations between the gingival phenotype, keratinized gingival width and the development or increase of gingival recession.

The presence of a thick gingiva is associated with wide keratinized tissue [35]. Likewise, whereas attached gingiva is regarded as beneficial for preserving optimal periodontal health, the importance of the width of keratinized tissue (WKT) in the maintenance of a satisfactory gingival condition is debatable. Provided good oral hygiene is implemented, the minimal WKT is compatible with gingival health [7]. Patients whose plaque control is suboptimal might encounter additional gingival recession [28]. However, optimal homecare may be a challenge in case of absence of KT [33]. The suggested amount of KT according to the American Academy of Periodontology is 2 mm, with 1 mm or more of attached gingival tissue [21]. Precise evaluation of the patients’ gingival phenotype is critical before, during and after orthodontic treatment as baseline recession, the thin gingival phenotype, lesser width of keratinized gingiva or thin symphysis have been reported to correlate significantly with the development or increase of gingival recession [14].

Although a number of studies have reported the correlation between periodontal variables and orthodontic parameters, no studies in the literature have evaluated the association between gingival recession height and width, clinical attachment level, width of keratinized tissue, gingival thickness on one hand and cephalometric parameters on the other. To the best of our knowledge there is no research to compare soft tissue dimensions with WITS. We hypothesized that there are differences in periodontal tissue of mandibular incisors between patients with different skeletal classes and vertical dimensions. The aim was to find correlations between measurements of the soft tissue surrounding mandibular incisors and selected parameters of cephalometric analysis.

## Materials and methods

### Study design

The study was planned as a prospective, cross-sectional trial. The material consisted of results of physical examinations and cephalograms obtained in the Department of Dental and Maxillofacial Radiology of the Medical University of Warsaw.

### Eligibility criteria

The participants for the study were recruited from patients of the Department of Orthodontics of Warsaw Medical University, who applied for treatment between January and December of 2015. The study was carried out in accordance with the Helsinki Declaration of 1975, as revised in Tokyo in 2004, after the study design was approved by the Bioethics Committee (KB/236/2014). Exclusion criteria were as follows: age under 18 years, past or current orthodontic therapy with fixed orthodontic appliances, maxillofacial and dental trauma, congenital and developmental defects of the head, medications affecting the periodontium, active gingival and periodontal diseases, restorations involving the cementoenamel junction, supernumerary or impacted teeth in the anterior area of the mandible, pregnancy or breastfeeding, and oral/labial piercing. Informed consent was obtained from all the individual participants included in the study.

### Physical examination

Clinical parameters were assessed by the same experienced and calibrated examiner (B.G.). Under local anesthesia the following parameters were evaluated:

- Gingival recession height (GRH)—the distance from the cementoenamel junction to the gingival margin evaluated mid-buccally.
- Gingival recession width (GRW)—the distance measured horizontally at the cementoenamel junction level from one border of the recession to the other.
- Gingival thickness (GT)—the bucco-palatal dimension measured 2 mm apically to the gingival margin by perpendicular insertion of a 10-mm endodontic spreader with a silicone stopper until the alveolar bone was reached. Each measurement was performed in triplicate to minimize inaccuracy.
- Width of keratinized tissue (WKT)—the distance between the most apical point of the gingival margin and the mucogingival junction evaluated mid-buccally. Mucogingival junction was demarcated by staining the mucogingival complex with iodine solution.
- Clinical attachment level (CAL)—the distance from the cementoenamel junction (CEJ) to the bottom of the gingival sulcus.

The GRH, GRW, WKT, and CAL were measured with the use of a millimeter-scaled periodontal probe (CPU 15 UNC, Hu-Friedy, Chicago, Illinois, USA) and rounded to the nearest 0.5 mm. An electronic caliper (YATO YT-7201, Toya, Wrocław, Poland) with 0.01 mm accuracy was used to assess the GT indicated on the endodontic reamer (Poldent, Warsaw, Poland) with a rubber stop.

A total of 6 patients with the thin gingival phenotype were recruited for the calibration exercise. The designated examiner recorded GRH, GWR, WKT, and CAL of the mandibular anterior teeth with an interval of 24 h between recordings. The calibration was accepted when ≥90% of the recordings could be reproduced within a difference of 1.0 mm and exact agreement was repeated in 75% of measurements.

### Radiological examination

Lateral cephalograms were performed by the same trained radiologist, in a natural head position, using Vatech Pax PCH-2500 (90 kV, 10 mA), at a distance of 2 m from the film. Radiographic examination was performed by an experienced and calibrated clinician (E.K.). An intraexaminer calibration was previously done by examining 10 nonstudy-related cephalometric images. The magnification of the images was estimated based on the scale placed in the X‑ray. The following parameters were assessed:

1. ANB—the angle between the lines: NA (nasion—point A) and NB (nasion—point B).
2. WITS—the distance between the perpendicular projection of points A and B on the functional occlusal plane.
3. NL:ML—the angle between the maxillary base plane (spina nasalis posterior-spina nasalis anterior) and mandibular base plane (a tangent to the lower border of mandible with the origin through menton).
4. 1-:ML—the angle between the long axis of most prominent incisor and the mandibular base plane (a tangent to the lower border of mandible with the origin through menton).
5. Symph. length—the distance from infradentale to gnation (perpendicular to the mandibular plane).
6. Symph. width—the longest distance from the buccal to labial cortex bone of the symphysis, perpendicular to symph. length.

### Statistics

Statistical analysis was performed with the use of Statistica 13. Descriptive statistics including the mean, standard deviation, median, minimum and maximum values were calculated for measured variables. Spearman’s correlation coefficient was calculated to determine a correlation between skeletal and dentoalveolar parameters. Subsequently, patients were classified according to ANB, WITS, ML:NL and 1‑:ML and differences between the means for each class of periodontal traits were compared with the use of the analysis of variance (ANOVA) and Tukey’s test. Moreover, clinical periodontal and radiological parameters were correlated. Criteria for the strength of correlation depending on Spearman’s correlation coefficient (R) were: R < 0.2 very weak, R 0.2–0.39 weak, R 0.4–0.59 moderate, R 0.6–0.79 strong, R > 0.8 very strong. When the *P* value was less than 0.05, the statistical test was determined to be significant. Finally, on the basis of obtained data, the sample size for further investigation was determined, considering the minimum 80% power value (t-test) and 95% confidence level.

## Results

### Sample characteristics

Of the 90 patients of both genders were invited to participate in the study, 63 patients met the eligibility criteria. Forty one patients signed informed consent for participation. Furthermore, 4 patients did not undergo cephalometric imaging and 2 were excluded because of low quality of radiological images. Overall, 35 patients aged 26.42 (±8.02) were enrolled. The study group involved 23 women (65%) and 12 men (34.3%). In all, 140 incisors were assessed.

### Clinical periodontal parameters

Gingival recession was found in 30 teeth (21.43%). In affected teeth the mean GRH was 1.7 mm and mean GRW was 2.16. GT was thin (≤1 mm) at 86 incisors and thick (>1 mm) at 54 incisors. The measurement of KG was below 2 mm in 6 teeth, 2 mm in 17 teeth, and over 2 mm in 117 teeth. CAL of 0 mm was found at 65 incisors and CAL exceeding 0 mm was found at 75 incisors. The results of clinical evaluation of periodontal tissues are shown in Table 1.

**Table 1:** Descriptive analysis of clinical parameters

	Measurements			
Variables	Mean [mm]	SD	Median [mm]	Range [mm]
GRH	0.37	0.81	0.00	0–3.5
GRW	0.41	0.89	0.00	0–3
GT	1.01	0.40	0.93	0.33–3.65
WKG	3.66	1.48	3.50	0–8
CAL	0.83	1.12	0.00	0–5

*SD* standard deviation, *GRH* gingival recession height, *GRW* gingival recession width, *GT* gingival thickness, *WKG* keratinized gingiva, *CAL* clinical attachment loss

### Cephalometric parameters

Table 2 shows data obtained in the cephalometric analysis. The analysis of correlation between dentoalveolar and skeletal parameters confirmed the existence of compensation (Table 3). There was a weak positive correlation between 1‑:ML and ANB, WITS and a negative correlation between 1:ML and ML:NL. A moderate positive correlation was found between symph. length and ANB, WITS, ML:NL. The latter correlations were statistically significant.

**Table 2:** Descriptive analysis of the cephalometric parameters

	Measurements				
Variables	Mean	SD	Median	Min	Max
ANB	2.61	2.99	3.1	−5.4	7.9
WITS	0.29	4.41	0.05	−14.7	7.4
ML:NL	24.29	5.81	23	13.4	44.6
1-:ML	95.12	9.61	97	68.9	109.3
Symph. length	30.51	3.68	30.07	23.14	42.84
Symph. width	13.68	1.47	13.55	11.31	16.54

*Symph. *symphysis, *SD* standard deviation, *Min* minimum, *Max* maximum

**Table 3:** Spearman’s correlation coefficient for skeletal and dentoalveolar parameters

Variables	1-:ML (p)	Symph. length (p)	Symph. width (p)
ANB	0.316 (0.064)	0.414 (0.013)**	0.000 (0.999)
WITS	0.215 (0.216)	0.414 (0.013)**	0.052 (0.766)
ML:NL	−0.269 (0.119)	0.472 (0.004)**	−0.297 (0.083)
1-:ML	1.000	0.057 (0.746)	0.216 (0.212)

**P < 0.05

*Symph. *symphysis

### Correlation between periodontal and cephalometric parameters

When averages of periodontal parameters were assessed in patient groups classified by ANB and WITS values, the only statistically significant differences were found for WKT in class II patients in comparison to class I and III patients according to ANB and also for WKT in class II and III patients according to WITS. Nevertheless, the tendency for more favorable periodontal condition (CAL, GT, GRW, GRH) in class II and less favorable in class III patients was noticeable. There were no differences in groups of patients divided according to ML:NL and 1‑:ML. There were statistically significant differences between groups divided according to symph. length (short in comparison to long for WKT; long in comparison to short and normal for GT) and symph. width (large in comparison to normal for WKT and narrow in comparison to normal and large for GT). Table 4 presents data for the above description.

**Table 4:** Periodontal parameters in the different skeletal groups

Classification (range)	*N* (%)	WKT	CAL	GT	GRW	GRH
*ANB*						
I (0–4°)	17 (48.57)	3.47a	0.67a	1.01a	0.38a	0.32a
II (>4°)	9 (25.71)	4.58b	0.86a	1.14a	0.43a	0.30a
III (<0°)	9 (25.71)	2.68a	1.18a	0.81a	0.71a	0.63a
*P*	–	0.004**	0.542	0.119	0.668	0.576
*WITS*						
I (−2 to 2 mm)	17 (48.57)	3.62ab	0.60a	1.02a	0.43a	0.34a
II (>2 mm$$)$$	9 (25.71)	4.42a	0.86a	1.15a	0.39a	0.30a
III (<−2 mm)	9 (25.71)	2.57b	1.34a	0.77a	0.66a	0.57a
*P*	–	0.008**	0.273	0.060	0.781	0.704
*ML:NL*						
Hypodivergent (<19°)	4 (11.43)	3.00a	0.00a	0.93a	0.00a	0.00a
Norm divergent (19–31°)	26 (74.29)	3.74a	0.87a	1.03a	0.50a	0.42a
Hyperdivergent (>31°)	5 (14.29)	3.80a	1.30a	0.97a	0.65a	0.40a
*P*	–	0.565	0.148	0.836	0.478	0.542
*1-:ML*						
Retrusion (<87°)	8 (22.86)	2.88a	0.88a	0.93a	0.25a	0.27a
Normal (87–101°)	15 (42.86)	3.85a	0.71a	0.99a	0.42a	0.32a
Protrusion (>101°)	12 (34.29)	3.95a	0.96a	1.09a	0.66a	0.51a
*P*	–	0.144	0.817	0.546	0.557	0.701
*Symph. length*						
Short (<26.77 mm)	4 (11.43)	2.28a	1.19a	0.83a	0.50a	0.53a
Normal (26.77–34.25 mm)	26 (74.29)	3.64ab	0.75a	0.97a	0.47a	0.38a
Long (>34.25 mm)	5 (14.29)	4.88b	0.95a	1.34b	0.40a	0.20a
*P*	–	0.007**	0.712	0.032**	0.983	0.785
*Symph. width*						
Large (>15.18 mm)	5 (14.29)	4.95b	0.20a	1.39b	0.00a	0.00a
Normal (12.19–15.18 mm)	23 (65.71)	3.28a	0.96a	0.95a	0.58a	0.47a
Narrow (<12.19 mm)	7 (20)	3.98ab	0.88a	0.93a	0.39a	0.32a
*P*	–	0.020**	0.323	0.015**	0.366	0.404

**P < 0.05

*a*, *b* describe the presence or absence of statistically significant differences. If means are designated with different letters (*a* and *b*) there is a statistically significant difference between them; if means are designated with the same letters (either *a* or *b*) the difference is not statistically significant

*GRW *gingival recession width, *GRH* gingival recession height, *GT* gingival thickness, *WKT* width of keratinized gingiva, *CAL* clinical attachment loss, *Symph. *symphysis

Table 5 presents Spearman’s correlation coefficient values for analyzed parameters. GRH, GRW and CAL did not correlate with any of cephalometric parameters. A statistically significant positive moderate correlation was found for GT and WITS and also symph. length. WKT correlated positively with ANB, WITS and symph. length and the correlation strength was moderate.

**Table 5:** Spearman’s correlation coefficient for periodontal and cephalometric parameters

Variables	ANB	WITS	ML:NL	1-:ML	Symph. width	Symph. length
*GRH*	−0.097	−0.087	0.064	0.093	−0.018	−0.125
*P* value	0.579	0.619	0.716	0.593	0.919	0.473
*GRW*	−0.079	−0.092	0.065	0.108	−0.002	−0.106
*P* value	0.652	0.601	0.709	0.539	0.993	0.543
*GT*	0.268	0.475	0.222	−0.027	0.214	0.450
*P* value	0.119	0.004**	0.201	0.877	0.251	0.025**
*WKT*	0.469	0.517	0.314	0.171	0.013	0.557
*P* value	0.004**	0.001**	0.066	0.326	0.943	0.001**
*CAL*	0.037	−0.041	−0.014	−0.046	−0.134	−0.121
*P* value	0.831	0.814	0.938	0.794	0.444	0.489

**P < 0.05

*GRW *gingival recession width, *GRH* gingival recession height, *GT* gingival thickness, *WKT* width of keratinized gingiva, *CAL* clinical attachment loss, *Symph. *symphysis

Variance of GT in this study was about 0.1. Assuming the difference for GT between two classes of WITS is equal to 0.15, the required sample size for further study is about 210 observation (three classes of WITS).

## Discussion

A number of predisposing anatomical and morphological factors for gingival recession have been found, such as alveolar bone dehiscence, gingival phenotype, narrow symphysis, and ectopic tooth eruption [25]. The aim of the present study was to assess the relation between GRH, GRW, WKT, GT, CAL and different cephalometric variables. Consequently, WKT was found to correlate with ANB, WITS and symphysis length, while GT was associated with WITS and symphysis length. Both WKT and GT are regarded as significant risk factors for gingival recession. Gingival recession may undermine outcomes of orthodontic treatment due to impaired dentofacial esthetics, psychological concerns or tooth hypersensitivity. The abovementioned issues relate directly to the age of the patient, as they are prone to be progressive in nature [27]. The need for orthodontic treatment steadily increases, which is why the evaluation of the gingival phenotype and the knowledge about predictors of gingival recessions is crucial as it may enhance the esthetic outcomes of orthodontic therapy.

For patients with adequate but less than optimal oral hygiene, who are undergoing orthodontic treatment, KT augmentation may be beneficial [1, 22]. By the same token, thick gingiva, together with the thick phenotype might contribute to minimizing the prevalence of advanced gingival recession following orthodontic treatment, as the thin phenotype is associated with thin underlying bone, bone dehiscence, and fenestration and reacts to insults and inflammation with gingival recession [18]. A positive correlation between WKT and GT has been underlined [35]. What is more, Mesen and Allais [25] proved a significant correlation between the width of KT, gingival phenotype and the development or increase of the gingival recession. Yared et al. [41] reported that recessions were more prevalent when the height of KT was <2 mm and GT <0.5 mm. Moreover, GT might be of more importance to recession than final inclination of incisors. Recently, a significant association has been found between mandibular incisor protrusion and the thin gingival phenotype [29].

Vasconcelos et al. [39] reported that reduction of the sagittal intermaxillary angle and retroclination of the lower incisors was correlated with the formation of more severe gingival recession. When ANB was less than 1.45° and 1‑:ML less than 92.6°, the possibility of developing recession increased four times. In our study GRH and GRW did not correlate directly with either ANB or WITS. Agreeing with results of Kaya et al. [19], we did not find relationship between GT and ANB, but GT correlated positively with WITS, which was not evaluated in their study. Also, in our research, patients with higher values of ANB and WITS presented better conditions in terms of WKT. Sperry et al. [37] compared orthodontically treated adults with Angle Class III to subjects with Angle Class I and Class II malocclusions and found that patients having excessive dental compensations in the Angle Class III group had thrice as many teeth with labial gingival recession. Several other studies, however, did not find the association between the WKT or GT and the Angle classification [20, 23, 42, 43]. As mentioned before, no previous study considered WITS.

While patients with the thin phenotype may demonstrate the highest risk of recession, the range of the lower incisors proclination that may contribute to recession development is debatable. Yet, Park et al. [29] reported more proclined incisors in the thin phenotype than in the thick phenotype for class III patients. According to a systematic review [14], more proclined teeth compared with less proclined teeth, with movement of the incisors outside the labial or lingual alveolar plate, which could lead to dehiscence and/or fenestration, might be at risk of developing gingival recession. It has been found that the final inclination of mandibular central incisors is more important than the total amount of proclination, as patients with inclination of more than 95° between long axis of incisors and mandibular plane showed greater gingival recession [41]. However, Renkema et al. [31] did not observe increased risk of developing gingival recession in proclined teeth during a 5-year observation period compared to nonproclined teeth. Similarly, another systematic review found no correlation between appliance-induced labial movement of mandibular incisors and gingival recession [4]. In cases of pronounced proclination of lower incisors without violating the osseous envelope of the alveolar process, there was no higher occurrence of gingival recession. It might be speculated that if the gingiva maintains appropriate thickness, it is more resistant and less affected by tension from large proclination. In that scenario the risk for formation of gingival recession could be significantly diminished. The relation of GR to retroclination of lower incisors, which is characteristic for class III patients is also unclear. Some of the authors report less favorable periodontal conditions and more frequent incidence of gingival recession in this group of patients [8]. Decreasing the 1:ML angle or the 1:NB distance were reported as risk factors of developing GR during orthodontic appliance therapy [5, 38]. On the other hand, Renkema et al. [30] did not find such a correlation.

Several studies established that symphysis morphology is associated with the quantity of the alveolar bone, as subjects with a long face and a steep mandibular plane had a narrower symphysis and thinner alveolar bone supporting anterior teeth compared to patients with a short face and a flat mandibular plane [11, 26, 32]. Due to this fact, patients with either lesser alveolar width or severe skeletal discrepancies demonstrate limitation in teeth movements and are most likely to exhibit loss of periodontal support during orthodontic therapy [3, 10]. In the study of Salti et al. [34] no correlation between facial length nor width and periodontal parameters (GR, CAL) was reported. Interestingly, they found a positive correlation between facial index values (ratio between height and width measurements) and periodontal destruction. Our results put this subject into another perspective, as we reported more favorable periodontal conditions (WKT, GT) in patients with long symphysis. However, it should be underlined that the external dimensions of the symphysis do not parallel the thickness of the alveolar bone. Consequently, some authors did not report any associations between symphysis shape and alveolar ridge changes after orthodontic treatment or gingival recession development [6, 24]. Thus, evaluation of symphysis morphology on cephalometric radiographs might not be a solid method aimed at predicting gingival recession in the anterior region of the mandible. Further studies are necessary to explain the association between the symphysis dimensions, alveolar width, periodontal tissues, and the risk of gingival recession.

## Limitations

First, it was a cross-sectional study and as such did not provide indications of the sequence of events. The sole purpose of this work was to examine and evaluate the relation between periodontal and cephalometric variables. Second, the sample of subjects was drawn from a pool of patients from one center, which may bias the findings. Finally, as it was a preliminary study, the study group could not be large enough to rule out all the confounders, but provided data to calculate the sample size for further investigation. Consequently, caution should be exercised when interpreting these results.

## Conclusion

In light of the ongoing discussion, our study supports evidence for an association between WKT and GT reflecting gingival phenotype morphology, and some cephalometric variables, namely ANB, WITS, and symphysis length. It is necessary to conduct a study on a larger sample of subjects to provide useful information regarding risk assessment and treatment concerns.
